# Development and validation of suicide crisis scale for international students in South Korea

**DOI:** 10.3389/fpsyg.2024.1417549

**Published:** 2024-09-20

**Authors:** Ki-Hyun Choi, Jung Hee Ha, Juliet Jue

**Affiliations:** ^1^Department of Multicultural Education, Hanyang University, Seoul, Republic of Korea; ^2^Graduate School of Counseling Psychology, Hanyang University, Seoul, Republic of Korea; ^3^Department of Art Therapy, Hanyang Cyber University, Seoul, Republic of Korea

**Keywords:** suicide crisis, suicidal thoughts, burdensomeness, maladjustment, social support

## Abstract

**Introduction:**

This study created and validated a scale to measure the level of suicide crisis among international students attending South Korean universities.

**Methods:**

Study 1 developed questions and constructed the components of the scale using an initial survey of 248 people and exploratory factor analysis. Study 2 validated the scale using a second survey of 340 participants and confirmatory factor analysis.

**Results:**

The results showed that 21 items were confirmed in six factors, which include lack of social support, burdensomeness, maladaptation to Korean culture, academic maladjustment, suicidal thoughts, and suicide risk.

**Discussion:**

We verified the convergent validity, criterion validity, and reliability of the scale. The significance and implications of this study are presented, along with suggestions for future research.

## 1 Introduction

The number of international students in Korea has increased from 86,000 in 2012 to 166,000 over the past 10 years (Ministry of Education, 2022). Over 50% are undergraduates, and others are graduate students or participating in language training. The increasing number of overseas students has both advantages and disadvantages (Lee, 2008). From the students’ perspective, studying abroad provides immersion in diverse educational opportunities and a new culture, the experience of a different way of life, and a broader perspective on global issues. In addition, students can develop language skills and create a global network. From the country’s perspective, foreign students contribute greatly to funding for higher education, spreading Korean culture, and raising Korea’s international status.

Studying abroad can be expensive, however, and adjusting to a new culture and foreign language can be challenging. Students may experience homesickness. Previous studies have noted international students who come to Korea experience emotional and behavioral problems related to losing their support system or feeling burdened by their studies (Baik, 2013; Han, 2009; Kim and Sohn, 2011). One survey of international students conducted by the Korea Institute for Health and Social Affairs (Kwak et al., 2021) found an average level of depression among international students at a level that requires professional therapeutic intervention. There were cases where students dropped out of school or even committed suicide (Kim, 2015; Lee and Kim, 2015). In a study among Korean international student counselors, all nine counselors who participated in the interview had counseled international student clients who had attempted suicide, and two of them reported that their clients’ suicide attempts led to death (Lee and Ha, 2023).

Although the condition and circumstances of each individual experiencing a suicidal crisis are diverse, it is also plausible that some common factors can be found in their psychological characteristics. People interpret their environment through a filtering system in their subjective world (Vuong, 2023). They filter their cultural and personal values in a complex manner and storing them within a system consisting of mindset, comfort zone, and environment (Nguyen et al., 2021). To understand an individual’s suicidal thoughts, it is important to analyze the cognitive information processing involved in how the individual absorbs, filters, and integrates emotions, beliefs and environmental factors.

Joiner (2005) identified that the psychological factors leading individuals to suicide include a sense of isolation and perceived burdensomeness. These two factors form conceptual framework: People receive various piece of information in their daily lives, and each individual interprets this information in their own way (Shannon, 1948). Shannon (1948) emphasized that in understanding suicidal thoughts, it is crucial to comprehend how individuals encode, transmit, and decode information related to emotional states, thoughts, and external inputs. The greater the uncertainty of the information, the more likely an individual is to focus excessively on it without efficiently filtering out what is unnecessary. This excessive focus on the negative aspects of information can lead to cognitive distortions and negative interpretations (Shannon, 1948), which are associated with suicidal thoughts (O’Connor and Nock, 2014; Van Orden et al., 2010).

Many international students who have difficulty adapting recognize that they need assistance or professional help to overcome psychological difficulties (Ding et al., 2021; Jeong, 2012), but they seldom receive support due to lack of information and language limitations (Keimyung University Student Counseling Center, 2019). These examples underscore the importance of screening and follow-up for international students. One support option is to develop tools to accurately assess students’ psychological states. Although scales to evaluate depression exist, a scale to evaluate the risk of suicide specifically for international students has not yet been developed.

The primary goal of evaluating suicide risk is to prevent suicide attempts and deaths. Early identification of individuals at risk allows for timely intervention and the provision of the support, resources, and treatment they need to overcome suicidal thoughts and impulses. A scale to evaluate suicide risk would also be helpful for the creation of university safety plans.

This study developed a suicide crisis scale tailored to the characteristics of international students. First, questions were developed and validated based on the common characteristics of all international students without distinguishing between their countries of origin. Second, module questions targeting students from specific countries were developed. Mun and Yoon (2021) reviewed research trends related to the adaptation of international students and found that out of a total of 571 papers, 427 focused on international students from specific countries. As the largest proportion of international students living in South Korea are Chinese, we derived cultural characteristics focusing on Chinese students residing in Korea.

## 2 Study 1: development of a suicide crisis scale for international students in Korea

We began by conducting a literature review to develop a suicide crisis scale for international students living in Korea. Additionally, we interviewed six students who had experienced a suicide crisis while studying abroad in Korea. The interviewees were selected through a pre-survey to ensure they were suitable for the study’s purpose. The pre-survey predominantly included the Suicidal Ideation Questionnaire (SIQ) (Reynolds, 1987) and the Center for Epidemiologic Studies Depression Scale (CES-D) (Radloff, 1977). International students who were considered to have experienced a suicidal crisis were defined by two criteria: a suicidal ideation score of 3 or higher on a 5-point scale in response to questions about their psychological crisis in Korea, or a depression score of 4 or higher on a 5-point scale. Using this approach, the initial items related to the experience of suicide crises among international students were identified through literature review and interviews.

We revised a total of 248 initial questions comprising six factors in two areas: direct suicide warning signs and potential risk factors. The direct crisis indicators include two factors, suicide risk and suicidal thoughts. Suicide risk is defined as a state in which a person is expected to commit suicide if given the opportunity. Suicidal thoughts are defined as thinking about suicide in daily life while not committing suicide right away (Kim and Kim, 2008). The thoughts may include considering specific means to commit suicide. Indirect suicidal crisis includes four factors that are closely related to suicide and/or depression: the lack of social support (Joeng et al., 2015; Kim and Kim, 2020; Kim et al., 2009; Park and Kim, 2018), perceived burdensomeness (Kang and Chang, 2018), academic maladjustment (Lee et al., 2023), and cultural maladjustment (Xiao and Ahn, 2016; Buyadaa and Yu, 2021).

Next, we collaborated with two multicultural counseling experts to narrow down the items to 175. Afterward, we verified content validity by surveying eight multicultural counselors working in college counseling centers to calculate the content validity ratio (CVR) and eliminated 40 questions, leaving 135 items. We created the original questionnaire in Korean, then translated to Chinese and English, using a reverse-translation process to ensure consistency. Finally, we conducted face validity verification with 13 foreign students enrolled in departments unrelated to counseling. They responded to questionnaires composed of the 135 extracted questions in Korean, English, and Chinese to determine whether the difficulty level of the questions was appropriate. Finally, a total of 135 questions were selected as preliminary questions.

### 2.1 Method

#### 2.1.1 Ethical considerations

The procedures for both Study 1 and Study 2 were approved by Hanyang University’s Institutional Review Board (HYUIRB-202312-016). We informed participants in advance that they may be psychologically affected while reading the questions because the questionnaire included suicide-related content. We made clear that participation in the survey was completely voluntary, and that there would be no disadvantage to those who chose not to participate. It was also explained that the anonymity of the responses was guaranteed and that data would be used only for the purpose of the study. After this information was presented, the participants signed the consent form if they agreed to participate in the study and began answering the questionnaire.

#### 2.1.2 Participants and procedure

The survey was conducted both face-to-face and online. For direct contact surveys, we visited three universities in Seoul with approval from their international student departments, providing the survey explanation and disclaimer verbally. For online surveys, we promoted the study via social media communities focused on international students and distributed online survey links in each language. On the first page of the survey link, the explanation of the study and the consent form were displayed in the form of an image, and participants continued to the questionnaire only if they voluntarily agreed to participate in the study. A total of 248 international students completed the survey. For the completed questionnaire, we performed data cleaning to remove errors, inconsistencies, and/or missing entries from the initial data. For example, if a question requiring only one response received multiple answers, it was treated as an error, and the data was excluded. Through this process, 33 responses were excluded, and data from 215 participants were ultimately used for analysis. The demographic information of the participants is presented in Table 1.

**Table 1:** Demographic information of the participants (*N* = 215).

Variable		Frequency	%	Variable		Frequency	%
Sex	Male	52	24.2	Level of Korean language	Beginner	53	24.7
	Female	163	75.8		Pre-intermediate	29	13.5
Age	<23	94	43.7		Intermediate	45	20.9
	23–25	65	30.2		Advanced	37	17.2
	26–29	32	14.9		Fluent	51	23.7
	29<	24	11.2				
Country of origin	China	127	59.0	Degree program	Undergraduate	132	61.4
	Vietnam	9	4.2		Master’s	63	29.3
	Indonesia	12	5.5		Doctoral	20	9.3
	France	7	3.3	Perceived economic status in Korea	Low	32	14.9
	Bangladesh	7	3.3		Middle – low	42	19.5
	Germany	6	2.8		Middle	106	49.3
	USA	6	2.8		Middle – high	30	14.0
	Uzbekistan	7	3.3		High	4	1.9
	etc.	34	15.8		N/A	1	0.5
Period of residence in Korea	<1 year	65	30.2	Financial source (multiple responses)	Scholarship	108	50.2
	1–3 years	69	32.1		Family support	164	76.3
	3–5 years	33	15.3		Part time job	49	22.8
	5–7 years	28	13.0		etc.	11	5.1
	7–10 years	8	3.7				
	10 years <	12	5.6				

#### 2.1.3 Data analysis

We conducted exploratory factor analysis using the maximum likelihood method of common factor analysis suggested by Jahng (2015) and direct Oblimin oblique rotation. The maximum likelihood method can quantify the goodness of fit of the factor model and correlation matrix, and the suitability of the model can be statistically verified. Oblique rotation is usually regarded as a more desirable method than orthogonal rotation in the behavioral sciences because it is realistic to assume that each latent variable has a certain level of correlation. While it is common to consider both the pattern matrix and the structure matrix when extracting factors through oblique rotation, we mainly used structure matrix based on Lee’s (1995) and Gorsuch’s (1983) suggestions.

Through exploratory factor analysis, we confirmed the assignment of questions to factors and removed questions that did not meet commonality and factor loading standards. We repeated this process for all questions until they met the criteria for commonality and factor loading. The process of selecting questions using exploratory factor analysis was conducted on all 215 responses and, separately, on responses from 127 Chinese students. Finally, we tested the reliability of the newly created scale.

### 2.2 Results

#### 2.2.1 Exploratory factor analysis results

##### 2.2.1.1 1st analysis: with the entire dataset

According to Kaiser (1974), the closer the value of the KMO index is to 1, the better. The standard is that if it is greater than 0.9, it is considered very good, if it is greater than 0.8, it can be considered good, and if it is greater than 0.6, it can be considered moderate. We found the KMO index of this scale was 0.87, confirming that each item was suitable for factor analysis (see Table 2). Bartlett’s test of sphericity was found to be significant at the 0.001 level. We determined the number of factors by considering the Eigenvalue, cumulative variance ratio, scree diagram, and factor loading, and extracted the final 6 factors. The number of factors exceeding 1, Kaiser’s (1974) Eigenvalue standard, was confirmed to be 6, and the cumulative variance ratio was 67.46%. In other words, the questions used in the exploratory factor analysis explained 67.46% of the six factors, meeting the 60% standard for fit (Kang, 2013).

**Table 2:** The values of the KMO index, Bartlett’s test of sphericity, the eigenvalue, and the cumulative variance ratio with all responses (*N* = 215).

Kaiser–Meyer–Olkin index		0.87
Bartlett’s test of sphericity	χ^2^	2807.50
	*df*	300
	*p*	0.000

Factor	1	2	3	4	5	6
Eigenvalue	8.35	2.68	1.97	1.63	1.20	1.04
Explained variance (%)	33.42	10.70	7.86	6.52	4.81	4.16
Cumulative variance (%)	33.42	44.11	51.97	58.49	63.30	67.46

The factor loadings of the questions in each factor are presented in Table 3. All factor loading values were greater than 0.40 and at least 0.10 greater than the factor loading value of other factors. In the process of creating preliminary questions theoretically and empirically, the established factors were named as follows: Factors one to six were named burdensomeness, academic maladjustment, lack of social support, suicide risk, maladaptation to Korean culture, and suicidal thoughts.

**Table 3:** Exploratory factor analysis results with all responses (*N* = 215).

Item number	Factor 1	Factor 2	Factor 3	Factor 4	Factor 5	Factor 6
	Burdensome-ness	Academic maladjustment	Lack of social support	Suicide risk	Maladaptation to Korean culture	Suicidal thoughts
42	0.91			0.44		−0.56
57	0.89			0.46		−0.58
43	0.58					
12	0.58		0.44			−0.43
70		0.90				
60		0.82				
10		0.56				
66			0.84			
79			0.74			
109			0.61			
4			0.55			−0.44
99	0.58			0.91		
123	0.53			0.88		
24				0.57		
45					0.75	
100					0.71	
50					0.67	
40					0.66	
80					0.64	
35					0.61	
113	0.61			0.47		−0.84
6	0.55			0.42		−0.83
39	0.56			0.51	0.42	−0.78
69	0.43			0.42		−0.67
129	0.45			0.43		−0.67

##### 2.2.1.2 2nd analysis: with the Chinese participants’ data only

The KMO index of this scale was 0.89, confirming that each item was suitable for factor analysis (see Table 4). In this study, the number of factors was determined by considering the cumulative variance ratio, scree plot, and factor loadings. We found the final six factors, while the number of factors exceeding Kaiser’s (1974) Eigenvalue criterion of 1 was confirmed to be 7. However, the cumulative variance ratio was 62.57% for 6 factors, meaning that the questions used in the exploratory factor analysis explained 62.57% of the six factors, again meeting the 60% standard (Kang, 2013).

**Table 4:** The values of the KMO index, Bartlett’s test of sphericity, the eigenvalue, and the cumulative variance ratio with Chinese participants’ data only (*N* = 127).

Kaiser–Meyer–Olkin index		0.87
Bartlett’s test of sphericity	χ^2^	2807.50
	*df*	300
	*p*	0.000

Factor	1	2	3	4	5	6
Eigenvalue	10.28	3.21	2.18	1.84	1.38	1.14
Explained variance (%)	32.11	10.03	6.81	5.76	4.30	3.55
Cumulative variance (%)	32.11	42.14	48.95	54.72	59.02	62.57

Finally, we confirmed the factor loadings of the questions for each factor. As presented in Table 5, factor loadings were greater than 0.40 and at least 0.10 greater than the factor loading value of other factors. The name of each factor was the same as for the entire sample.

**Table 5:** Exploratory factor analysis results with Chinese participants’ data only (*N* = 127).

Item number	Factor 1	Factor 2	Factor 3	Factor 4	Factor 5	Factor 6
	Suicidal thoughts	Academic maladjustment	Lack of social support	Suicide risk	Maladaptation to Korean culture	Burdensome-ness
113	0.85			0.44		
6	0.81		0.41			
39	0.75			0.49	0.41	
69	0.70					
82	0.69					
129	0.68			0.40		
70		0.85				
60		0.82				
10		0.61				
30		0.57				
20		0.46				
66			0.83			
79			0.75			
119	0.45		0.60			−0.43
109			0.58			
4	0.42		0.57			
99	0.49			0.89		−0.57
123	0.51			0.87		−0.51
24				0.58		
50					0.73	
45					0.68	
40					0.68	
100		0.43			0.67	
65					0.66	
110					0.65	
35					0.64	
80					0.61	
75					0.45	
42	0.57			0.41		−0.91
57	0.58			0.42		−0.89
43						−0.57
12	0.41			0.44		−0.57

#### 2.2.2 Reliability analysis results

The results of the reliability analysis are presented in Table 6. Cronbach’s *α* was calculated to confirm internal consistency for each factor, and all factors’ reliability coefficients were above 0.70.

**Table 6:** Reliability analysis results.

		Cronbach’s *α*	
		All responses (*N* = 215)	Chinese participants (*N* = 127)
Potential risk factors	Lack of social support	0.77	0.80
	Burdensomeness	0.84	0.84
	Maladaptation to Korean culture	0.83	0.86
	Academic maladjustment	0.80	0.82
Direct crisis indicators	Suicide risk	0.82	0.82
	Suicidal thoughts	0.88	0.89
International students’ suicide crisis		0.91	0.93

#### 2.2.3 Items extracted from study 1

Based on the analysis of the survey data, basic questions applicable to all international students regardless of country of origin were extracted into 25 questions in 6 factors. Four questions each were extracted from the lack of social support and the feeling of being a burden on others. Three questions were extracted for academic maladaptation, and six for Korean cultural maladaptation. Five questions were extracted for suicidal thoughts, and three for suicide risk.

For Chinese international students only, an additional seven questions were extracted from four factors: one for the lack of social support, one for suicidal thoughts, two for academic maladjustment, and three for Korean culture maladaptation.

## 3 Study 2: validation of the suicide crisis scale

### 3.1 Method

#### 3.1.1 Participants and procedure

The procedure used in Study 2 was the same as in Study 1, only the participants were new. A total of 340 international students in Korea participated in this survey. Out of 340 responses, 32 incomplete responses were removed, and 308 responses were analyzed. Participants’ demographic information is presented in Table 7.

**Table 7:** Demographic information of the participants (*N* = 308).

Variable		Frequency	%	Variable		Frequency	%
Sex	Male	74	24.0	Level of Korean language	Beginner	85	27.6
	Female	234	76.0		Pre-intermediate	38	12.3
Age	<23	130	42.2		Intermediate	63	20.5
	23–25	93	30.2		Advanced	53	17.2
	26–29	44	14.3		Fluent	69	22.4
	29<	41	13.3				
Country of origin	China	159	51.6	Degree program	Undergraduate	191	62.0
	Vietnam	11	3.6		Master’s	88	28.6
	Indonesia	18	5.8		Doctoral	28	9.1
	France	14	4.5		N/A	1	0.3
	Japan	14	4.5	Perceived economic status in Korea	Low	40	13.0
	Malaysia	9	2.9		Middle – low	65	21.1
	USA	11	3.3		Middle	147	47.7
	Uzbekistan	7	2.3		Middle – high	41	13.3
	Etc	65	21.1		High	15	4.9
Period of residence in Korea	<1 year	110	35.7	Financial source (multiple responses)	Scholarship	137	51.3
	1–3 years	93	30.2		Family support	196	73.4
	3–5 years	45	14.6		Part time job	67	25.1
	5–7 years	30	9.7		etc.	26	9.7
	7–10 years	11	3.6				
	10 years <	19	6.2				

#### 3.1.2 Measures

We used six measures to validate the suicide crisis scale (SCS), ensuring the measures were validated using statistical analysis methods at the time of development, and that the concept measured by the factor was similar or opposite to the sub-concept of the SCS and suitable for use as a reference.

##### 3.1.2.1 The Scale for Suicidal Ideation

The Scale for Suicidal Ideation (Beck et al., 1979) is a single factor scale consisting of 19 questions. Each situation is presented as a question and participants are asked to respond with a score between 0 and 2 points. The higher the score, the more frequently people think about suicide and the more likely they are to commit suicide. The scale’s reliability coefficient was found to be 0.99 in this study.

##### 3.1.2.2 The Acquired Capability for Suicide Scale

The Acquired Capability for Suicide Scale was originally developed by Van Orden et al. (2008). This scale consists of 20 total items divided into two sub-factors: fearlessness about death and habituation to pain. We used the former only. A higher score means less fear of committing suicide, which increases the risk of implementation. The reliability score was 0.94 in this study.

##### 3.1.2.3 Rosenberg self-esteem scale

We chose self-esteem as the opposite of burdensomeness. They are two psychological constructs that relate to how individuals perceive themselves and their role in relationships, but they represent different ends of the spectrum. High self-esteem generally contributes to positive outcomes, while feelings of burdensomeness tend to have negative consequences. Rosenberg (1965) developed the Self-Esteem Scale, which is composed of 10 items: five questions with positive content and five with negative. After reverse scoring the negative items, all scores are added, with higher scores indicating that people perceive themselves as valuable and positive persons. Cronbach’s *α* was 0.97 in this study.

##### 3.1.2.4 The Acculturative Stress Scale for international students

To examine international students’ adjustment stress, we used the Acculturative Stress Scale developed by Sandhu and Asrabadi (1994). This scale has 36 items, comprising six sub-variables: perceived discrimination, homesickness, fear, guilt, perceived hatred, and stress due to change (cultural shock). This study selected six out of nine questions from the perceived discrimination sub-scale to ensure criterion validity of our study. We excluded three items regarding appearance differentiation such as skin color, as they did not apply to Chinese students in Korea. The higher the final score, the greater the perception of discrimination from those around a person living in a different culture. Cronbach’s *α* for this scale was 0.90 in this study.

##### 3.1.2.5 Measuring Adjustment to College Questionnaire

To measure students’ perceived adjustment levels, we used the Measuring Adjustment to College Questionnaire developed by Baker and Siryk (1989). This scale consists of four sub-factors: academic adjustment, social adjustment, personal-emotional adjustment, and institutional attachment. The higher the score, the better the student has adapted to overall college life in a different culture. Internal consistency, measured by Cronbach’s *α*, was 0.97 in this study.

##### 3.1.2.6 The multidimensional scale of perceived social support

This scale was developed by Zimet et al. (1988) to evaluate three sources of support: family, friends, and significant others. To use it as a reference for the lack of social support on the international student SCS, we excluded four questions about family support that did not fit the purpose of the study and used the remaining questions. Cronbach’s α was 0.95 in this study.

#### 3.1.3 Analysis method

We conducted validation of the two scales for the full sample of international students and for the sample of Chinese students only. Confirmatory factor analysis was conducted to verify convergent validity, and the final items of the SCS were derived. Next, we analyzed descriptive statistics, including skewness and kurtosis, to verify normality assumptions. In addition, correlation analysis was conducted to verify criterion validity. Finally, we calculated the reliability coefficient for each factor of the SCS.

### 3.2 Results

#### 3.2.1 Confirmatory factor analysis results

To validate the SCS, we conducted confirmatory factor analysis with the entire dataset and with Chinese students’ data, respectively.

##### 3.2.1.1 1st analysis: with the entire dataset

An initial confirmatory factor analysis was conducted to assign the 26 items extracted through exploratory factor analysis to tentative factors. The initial model’s goodness of fit was found to be appropriate. However, in the process of calculating convergent validity, we found that the results of average variance extracted (AVE) fell short of the standard. The lack of social support’s AVE value was 0.49, and the maladaptation to Korean culture’s AVE value was 0.46. Thus, we removed items that seemed to reduce validity. For instance, an item included in the suicidal ideation factor (“I have tried to commit suicide, but I have stopped myself before actually doing it”) was deleted as it was judged to deviate from the concept of suicidal ideation. Finally, a model consisting of a total of 21 questions was established and used in analysis after confirming the suitability of the model (see Table 8).

**Table 8:** The fit of confirmatory factor analysis with all responses.

	*x*^2^	*df*	TLI	CFI	RMSEA (90% CI)	SRMR
Initial model (25 items)	585.44^***^	260	0.90	0.92	0.06 (0.057–0.071)	0.06
Final model (21 items)	304.26^***^	174	0.95	0.96	0.05 (0.040–0.058)	0.06

***p < 0.001.

**Figure 1:**
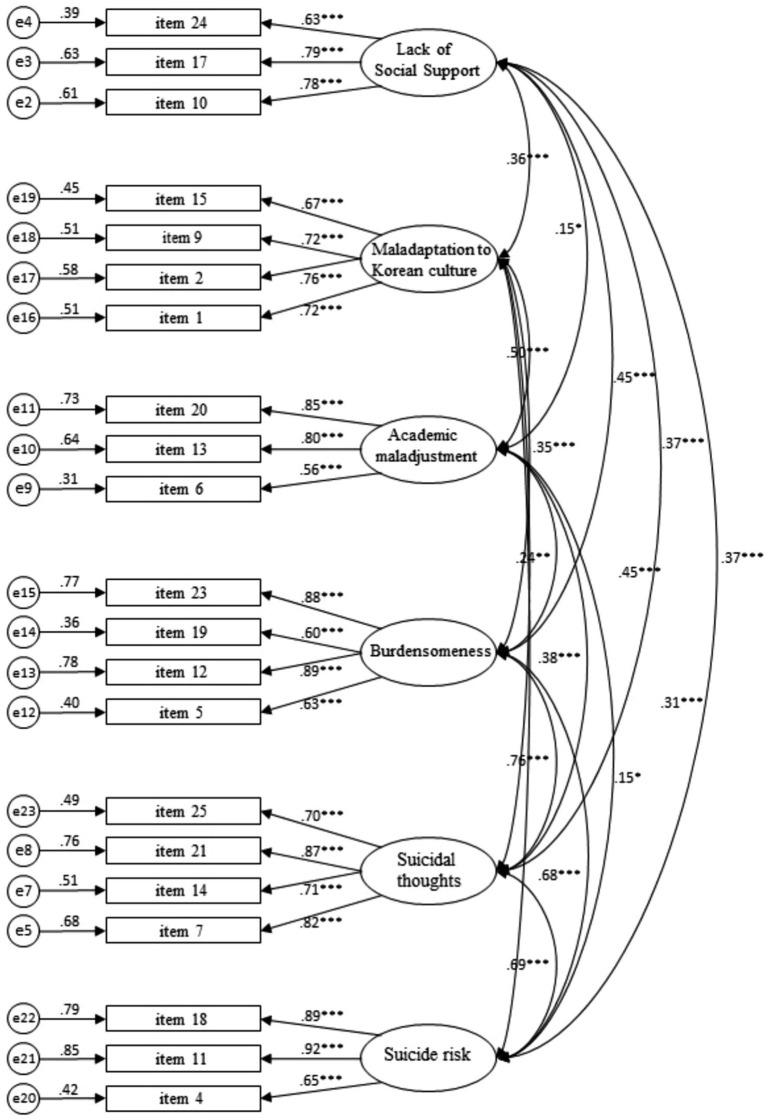
Confirmatory factor analysis results with all responses.

As presented in Table 9 and Figure 1, the standardized coefficients for all questions in the final model have values of 0.50 or higher. For the lack of social support factor, AVE was 0.54 and CR was 0.78, meeting the condition of convergent validity. For maladaptation to Korean culture, the AVE value was 0.51, and CR was 0.81. All other factors met the conditions of convergent validity.

**Table 9:** Confirmatory factor analysis results with all responses.

Path between variables		*Β*	*β*	Standard error	Critical ratio	Average variance extraction	Concept reliability
Lack of social support	→ #10	1	0.78^***^			0.54	0.78
	→ #17	1.09	0.79^***^	0.10	11.00		
	→ #24	0.99	0.63^***^	0.10	9.71		
Maladaptation to Korean culture	→ #1	1	0.72^***^			0.51	0.81
	→ #2	1.03	0.76^***^	0.09	11.34		
	→ #9	0.86	0.72^***^	0.08	10.88		
	→ #15	0.93	0.67^***^	0.09	10.29		
Academic maladjustment	→ #6	1	0.56^***^			0.56	0.79
	→ #13	1.60	0.80^***^	0.17	9.19		
	→ #20	1.64	0.85^***^	0.18	9.18		
Burdensomeness	→ #5	1	0.63^***^			0.58	0.84
	→ #12	1.82	0.89^***^	0.15	12.17		
	→ #19	0.92	0.60^***^	0.10	9.16		
	→ #23	1.71	0.88^***^	0.14	12.14		
Suicide risk	→ #4	1	0.65^***^			0.69	0.87
	→ #11	1.90	0.92^***^	0.15	13.01		
	→ #18	1.87	0.89^***^	0.15	12.82		
Suicidal thoughts	→ #7	1	0.83^***^			0.61	0.86
	→ #14	0.88	0.71^***^	0.07	13.56		
	→ #21	1.03	0.87^***^	0.06	17.70		
	→ #25	0.78	0.70^***^	0.06	13.28		

***p < 0.001.

##### 3.2.1.2 2nd analysis: with the Chinese participants’ data only

Seven questions used only in the Chinese international student sample were added to the final 21 questions from the entire international student sample, resulting in a total of 28 questions used in the initial factor analysis. We conducted the analysis in the same manner as for the full dataset. Three items were found to impair the goodness of fit and were removed from the final model. After confirming the suitability of the confirmatory factor analysis of the final model (see Table 10), we conducted confirmatory factor analysis.

**Table 10:** The fit of confirmatory factor analysis with Chinese participants’ data only.

	*x*^2^	*df*	TLI	CFI	RMSEA (90% CI)	SRMR
Initial model (28 items)	639.84^***^	335	0.86	0.87	0.08 (0.067–0.085)	0.86
Final model (25 items)	454.09^***^	260	0.89	0.91	0.07 (0.058–0.079)	0.08

***p < 0.001.

**Figure 2:**
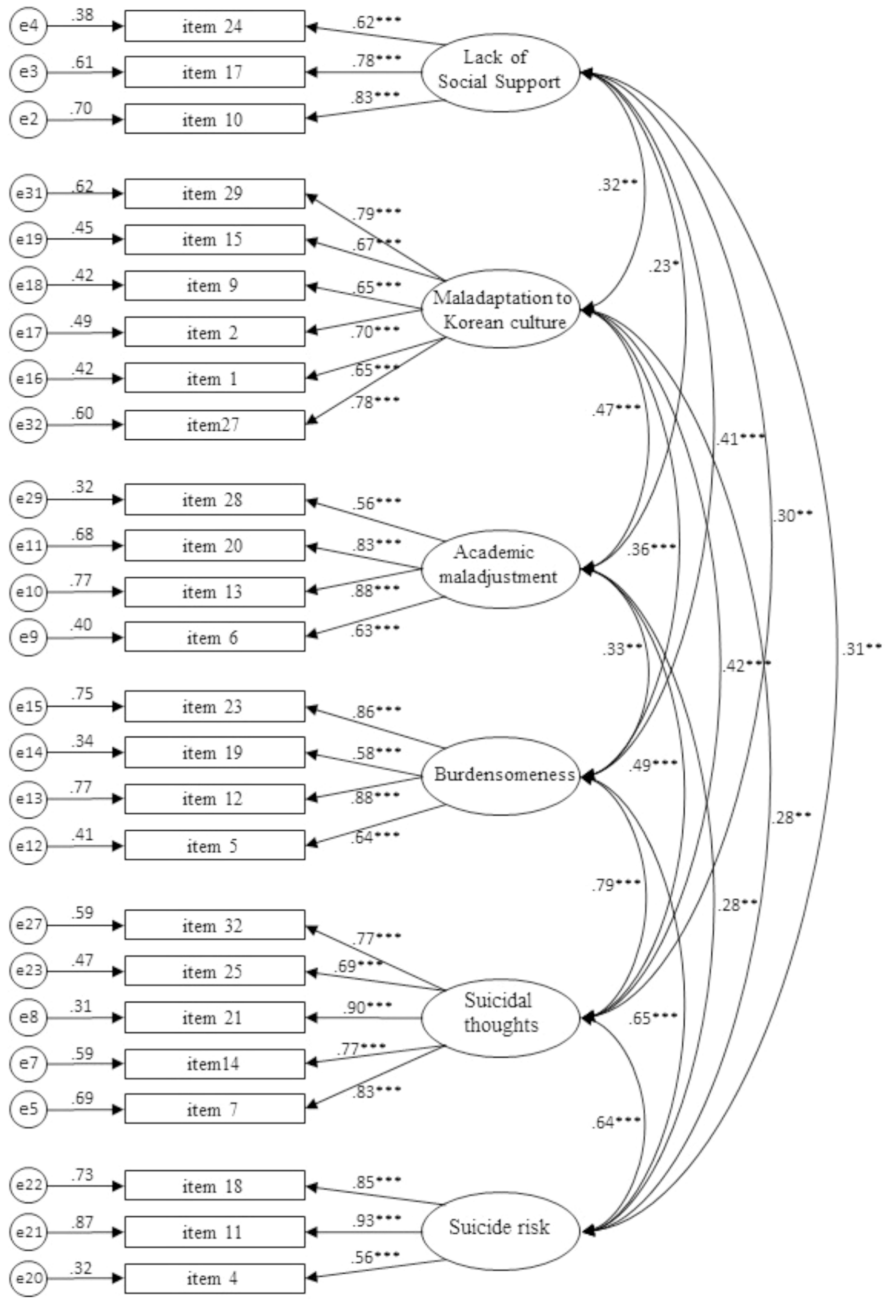
Confirmatory factor analysis results with Chinese participants’ data only.

Table 11 and Figure 2 show the confirmatory factor analysis results. All items have standardized coefficients of 0.50 or higher. Through examining AVE values and CR values, we confirmed that all the factors in the final model have sufficient levels of convergent validity.

**Table 11:** Confirmatory factor analysis results with Chinese participants’ data only.

Path between variables		*Β*	*β*	Standard error	Critical ratio	Average variance extraction	Concept reliability
Lack of social support	→ #10	1	0.83^***^			0.56	0.79
	→ #17	0.96	0.78^***^	0.12	8.30		
	→ #24	0.88	0.62^***^	0.12	7.14		
Maladaptation to Korean culture	→ #1	1	0.65^***^			0.50	0.86
	→ #2	1.16	0.70^***^	0.16	7.38		
	→ #9	0.94	0.65^***^	0.14	6.92		
	→ #15	1.10	0.67^***^	0.15	7.14		
	→ #27	1.32	0.78^***^	0.17	7.96		
	→ #29	1.35	0.79^***^	0.17	8.04		
Academic maladjustment	→ #6	1	0.63^***^			0.54	0.82
	→ #13	1.56	0.88^***^	0.19	8.23		
	→ #20	1.40	0.83^***^	0.17	8.06		
	→ #28	0.91	0.56^***^	0.15	6.05		
Burdensomeness	→ #5	1	0.64^***^			0.57	0.84
	→ #12	1.88	0.88^***^	0.21	8.83		
	→ #19	0.87	0.58^***^	0.14	6.42		
	→ #23	1.80	0.86^***^	0.21	8.74		
Suicide risk	→ #4	1	0.56^***^			0.64	0.84
	→ #11	2.79	0.93^***^	0.37	7.55		
	→ #18	2.73	0.85^***^	0.37	7.44		
Suicidal thoughts	→ #7	1	0.83^***^			0.63	0.89
	→ #14	0.94	0.77^***^	0.09	11.07		
	→ #21	1.08	0.90^***^	0.08	14.02		
	→ #25	0.77	0.69^***^	0.08	9.51		
	→ #32	0.92	0.77^***^	0.08	11.07		

***p < 0.001.

#### 3.2.2 The final items on the SCS

The final questions included in the SCS are presented in Table 12. There are 21 questions in six factors applicable to all international students regardless of country of origin. In addition, four questions extracted from three factors are applicable to Chinese students.

**Table 12:** The final items on the SCS.

	Factor	Item
1	Lack of social support	I feel warmth and affection from people close to me.
		I feel that many people genuinely care about me.
		I feel that there are people who will offer assistance when I am struggling.
	Burdensomeness	I believe people around me would be happier without me.
		If given the choice, it would have been better not to have been born at all.
		I feel that I am making things worse for those around me.
		I wish I had never been born.
	Maladaptation to Korean culture	I feel that I have been unfairly disadvantaged in group projects because I am not Korean.
		When collaborating on group projects with Korean students, I perceive subtle cues of being regarded as inferior.
		As a research assistant in a laboratory, I subtly feel alienated due to my non-Korean background.
		I feel discriminated against while studying in Korea.
		I find it difficult to establish friendships with Koreans as the culture of my home country is subtly belittled or disrespected in Korea.*
		It is hard to form close relationships with Koreans as they maintain boundaries with non-Koreans.*
	Academic maladjustment	In addition to adjusting to the Korean language, I feel overwhelmed by the demands of managing a heavy workload.
		When working on group projects with Korean students, I feel that my limited proficiency in Korean as a challenge.
		I feel that my limited proficiency in Korean hinders my academic work in Korea.
		I feel overwhelmed trying to keep up with my studies in Korea (or in Korean labs) due to Korea’s “Fast-paced (ppalli-ppalli)” culture.*
2	Suicidal thoughts	I have contemplated the least difficult method of suicide that I could act.
		I have contemplated the possibility of being killed in a major accident.
		I have imagined scenarios where I commit suicide.
		I have considered what would I include in my will if I were to commit suicide.
		I have considered how others would feel if I were to die by suicide.*
	Suicide risk	I intend to die by suicide when the opportunity arises.
		If my situation does not improve, I would die by suicide.
		I believe I would die by suicide if the opportunity arose.

1: Potential risk factors; 2: Direct crisis indicators, *additional items applicable to Chinese students.

#### 3.2.3 Correlation analysis with criterion scales

In order to examine the criterion validity of each factor in the SCS, we conducted correlation analysis between sub-scales of the SCS and applicable criterion scales. Statistically significant correlation results in such analysis would demonstrate criterion sufficient validity (Seong, 2014).

##### 3.2.3.1 1st analysis: with the entire dataset

Table 13 presents the results of correlation analysis between the criterion scales and the SCS sub-scales. The lack of social support showed a strong negative correlation with social support, *r* = 0.80 (*p* < 0.01). Burdensomeness was negatively correlated with self-esteem, *r* = 0.81, (*p* < 0.01), which was set as a reference. In the case of other scales, correlation analysis with the scale set as the criterion showed statistically significant results.

**Table 13:** Correlation between sub-scales of the SCS and applicable criterion scales: with the entire dataset (*N* = 308).

Variable	Perceived social support	Self-esteem	Acculturative stress	Adjustment to college	Beck’s suicidal ideation	Acquired capability for suicide
Suicide risk	−0.41^**^	−0.59^**^	0.65^**^	−0.53^**^	0.76^**^	0.29^**^
Lack of social support	−0.80^**^	−0.37^**^	0.27^**^	−0.13^*^	0.26^**^	0.16^**^
Burdensomeness	−0.32^**^	−0.81^**^	0.35^**^	−0.28^**^	0.64^**^	0.31^**^
Maladaptation to Korean culture	−0.22^**^	−0.17^**^	0.78^**^	−0.32^**^	0.39^**^	0.15^*^
Academic maladjustment	−0.09	−0.14^*^	0.41^**^	−0.87^**^	0.28^**^	0.01
Suicidal thoughts	−0.26^**^	−0.50^**^	0.39^**^	−0.31^**^	0.89^**^	0.27^**^
Total Suicide Crisis	−0.25^**^	−0.55^**^	0.28^**^	−0.12^*^	0.57^**^	0.58^**^

*p < 0.05, **p < 0.01.

##### 3.2.3.2 2nd analysis: with the Chinese participants’ data only

The correlation analysis results with Chinese participants’ data are presented in Table 14. Lack of social support was negatively correlated with social support, the criterion variable, *r* = 0.83 (*p* < 0.01). Burdensomeness showed a strong negative correlation with self-esteem, *r* = 0.89 (*p* < 0.01). Maladaptation to Korean culture presented a high positive correlation with acculturation stress, *r* = 0.79 (*p* < 0.01), while academic maladjustment had a strong negative correlation with college life adaptation, *r* = 0.90 (*p* < 0.01). Suicidal thoughts showed a strong positive correlation with Beck’s suicidal ideation scale, *r* = 0.89 (*p* < 0.01), and suicide risk showed a strong positive correlation with acquired capability for suicide, *r* = 0.29 (*p* < 0.01). Therefore, we confirmed that all the test variables showed strong correlations with criterion scales.

**Table 14:** Correlation between sub-scales of the SCS and applicable criterion scales: with the Chinese participants’ data only (*N* = 159).

Variable	Perceived social support	Self-esteem	Acculturative stress	Adjustment to college	Beck’s suicidal ideation	Acquired capability for suicide
Suicide risk	−0.44^**^	−0.65^**^	0.64^**^	−0.55^**^	0.82^**^	0.26^**^
Lack of social support	−0.83^**^	−0.38^**^	0.27^**^	−0.18^*^	0.23^**^	0.18^*^
Burdensomeness	−0.33^**^	−0.89^**^	0.32^**^	−0.28^**^	0.59^**^	0.27^**^
Maladaptation to Korean culture	−0.25^**^	−0.11	0.79^**^	−0.20^*^	0.33^**^	0.07
Academic maladjustment	−0.17^*^	−0.18^*^	0.36^**^	−0.90^**^	0.37^**^	0.01
Suicidal thoughts	−0.24^**^	−0.58^**^	0.37^**^	−0.36^**^	0.94^**^	0.22^**^
Total suicide crisis	−0.24^**^	−0.51^**^	0.29^**^	−0.19^*^	0.57^**^	0.60^**^

*p < 0.05, **p < 0.01.

#### 3.2.4 Reliability analysis

Results of the reliability analysis on six factors consisting of questions derived from the entire sample and the Chinese sample are presented in Table 15. Cronbach’s *α* coefficients were calculated to confirm internal consistency for each factor. The factor with the lowest reliability coefficient was the *lack of social support*; it was 0.77 in the overall sample and 0.78 in the Chinese sample. The reliability coefficient of international students’ suicidal crisis experience for all questions was 0.91 in the entire sample and 0.91 in the Chinese sample.

**Table 15:** Reliability analysis results.

		Cronbach’s *α*	
		All responses (*N* = 308)	Chinese participants (*N* = 159)
Potential risk factors	Lack of social support	0.77	0.78
	Burdensomeness	0.84	0.83
	Maladaptation to Korean culture	0.81	0.86
	Academic maladjustment	0.77	0.82
Direct crisis indicators	Suicide risk	0.86	0.81
	Suicidal thoughts	0.86	0.89
International students’ suicide crisis		0.91	0.91

## 4 Discussion

This study examined the characteristics of suicide crisis that foreign students in Korea may experience and developed and validated a suicide crisis scale to reflect the characteristics. The results, implications, and significance of this study are as follows.

First, we produced 175 preliminary questions through a literature review and in-depth interviews with 6 international students, which we narrowed down to 135 preliminary questions consisting of 7 factors through expert evaluation. After conducting a first survey with 215 international students, we performed exploratory factor analysis and extracted 6 factors and 32 questions. The second survey was conducted with 308 international students, and confirmatory factor analysis was performed to produce six factors and 21 final questions, for which we verified the normality assumption, criterion validity, and reliability.

The suicide crisis scale for international students consists of six factors: suicide risk, suicidal thoughts, lack of social support, burdensomeness, maladaptation to Korean culture, and academic maladjustment. The first factor, *suicide risk*, is an important factor in that it measures the level of crisis related to suicide. International students are more likely than students at their home universities to experience psychological problems just by living their daily lives in an unfamiliar place (Poyrazli et al., 2004; Thomas and Choi, 2006). Therefore, directly measuring the possibility of suicide attempts among international students is useful in that it allows for determination of severity and enables the provision of counseling intervention before students actually attempt suicide.

Second, the *suicidal thoughts* factor measures the crisis level of international students’ experiences of thinking about or imagining death. It measures fantasies about death in everyday stressful situations rather than a crisis level that could lead to a suicide attempt at any time, so it is useful as a measure of a broader sense of suicide crisis. Although college students with suicidal thoughts experience both suicide thoughts due to stressful situations and the desire to live well (Jeong and Shim, 2022), people who have thoughts of suicide are 47 times more likely to die from suicidal behavior than people who do not have thoughts of suicide (Kim et al., 2015). Since persistent suicidal thoughts constitute a risk factor for suicide plans and suicide attempts (Im and Jung, 2002), their evaluation can be an important part of suicide prevention.

Third, the *lack of social support* factor measures the degree to which one feels that he or she does not receive social support from the people around him or her. Joiner (2005) suggested thwarted belongingness as a decisive factor leading to suicide. Interpersonal relationships have been found to have a significant impact on suicidal thoughts (Jeong and Shim, 2022; Lee, 2015; Park and Kim, 2017; Park and Lee, 2021; Yoon and Lee, 2022), including among foreign students. Given that students who study abroad are faced with the task of creating a whole new social network (Pavlacic et al., 2023), the lack of social support is an important factor in measuring the experience of suicide crisis among international students.

Today’s world, often referred to as the infosphere, is a space where online and offline information are intertwined and mutually influential. The infosphere impacts how individuals and societies process, share, and interpret information, shaping public opinion, personal beliefs, and even decision-making processes. Although the questions used in the SCS scale did not specifically address the influence of internet use on interpersonal relationships or daily life, we were able to infer such influences during in-depth interviews. For instance, when asked about experiences of cultural differences and discrimination, not a few Chinese students expressed shock at seeing negative sentiments toward China expressed online by Koreans, particularly regarding the increase in negative views of China due to COVID-19. As a result, they mentioned concerns that professors or other colleagues in their labs might dislike or discriminate against them because of their nationality. Through these responses, we were able to implicitly conclude that the infosphere does indeed influence individuals. If future research examines the relationship between suicidal thoughts and the flow of information across different platforms and mediums, including the internet, social media, traditional media, and interpersonal communication, we may be able to confirm the effect of the infosphere on suicidal ideation.

The fourth factor is *burdensomeness*, which measures the degree to which one perceives oneself as a nuisance or as being useless to the environment or people around them. International students experiencing suicidal thoughts may develop a strong sense of non-belonging and perceived burdensomeness through their personal cognitive interpretation of their experiences while studying abroad in Korea—conclusions rooted in their core beliefs and values. If the information they absorb strongly supports negative self-perceptions or perceived burdensomeness, this could dominate their cognitive landscape, pushing them toward suicidal thoughts (Vuong, 2023). In addition, mindsponge theory suggests that individuals selectively absorb information that aligns with or challenges their core beliefs. A person contemplating suicide may focus selectively on negative information, reinforcing feelings of hopelessness and despair. This cognitive filtering might exclude positive or hopeful messages, making it difficult for them to perceive alternatives to suicide.

Perceiving oneself as a burden to others is an important contributing factor to suicidal behavior (Joiner, 2005). One study among American college students found that frustrated belongingness and perceived burdensomeness were positively correlated with the acquisition of self-harm skills (Davidson et al., 2010). For Asian American college students, burdensomeness more strongly predicts suicidal thoughts, and thwarted belongingness moderates the relationship between burdensomeness and suicidal thoughts (Wong et al., 2011). Similarly, Baik (2011) found that among international students, those with higher self-esteem adapted better to university life. In other words, the more positive the sense of self-worth, the better the student adapted to college life, while the more negative the student’s sense of self-worth, the more difficult it was for them to adapt to college life, and in serious cases, these challenges can lead to suicidal thoughts or actions.

The fifth factor is *maladaptation to Korean culture*, which measures the degree of cultural differences or shock, and/or discrimination experienced by international students in Korea. Foreign students experience cultural discrimination while studying abroad and may become psychologically unstable due to their inability to adapt to the dominant culture (Lee and Park, 2019; Yu et al., 2014); they also often experience a lot of stress related to acculturation (Lee and Lee, 2021; Zhou et al., 2023). One study among counselors who worked with international students found cultural barriers and situational problems—including daily adaptation to environmental differences, discrimination and prejudice experienced at university, and environmental and economic issues—to be one of two primary categories of difficulties (Oh and Lee, 2018). Therefore, for international students, cultural adaptation can be considered an important factor contributing to a satisfactory study abroad experience, and cultural maladaptation can be seen as a factor that increases the risk of suicide.

Finally, the sixth factor is *academic maladjustment*, which addresses the degree of academic achievement of international students at Korean universities. Oh and Lee (2018) identified academic difficulties, including language barriers, problems in basic academic skills, and lack of confidence in their academic field, as a primary driver of stress among international students. International students experience stress because they need to have high-level language skills to perform academic tasks (Jin and Lim, 2021); they may also experience stress as they compete with local students (Lim and Lee, 2017). Such academic maladjustment leads to various psychological problems (Kim and Kim, 2014; Lee et al., 2023; Park, 2022), and in severe cases, can lead to academic interruption or suicide attempts (Jin and Chi, 2017; Lee and Ha, 2023; Shin et al., 2018). Therefore, academic maladjustment factors can be considered an important factor in measuring the suicide risk of international students.

The significance of this study is as follows. First, it addresses a gap in studies examining suicide crisis among international students with structured questions. The results of this study offer a foundation for approaches to addressing suicide crisis among international students. Second, the sub-factors of our scale reflect the specific characteristics of suicide crisis. In addition to direct suicide crisis and suicidal thoughts, we added important factors that could potentially predict suicide crisis of foreign students. Lack of social support and burdensomeness were empirically demonstrated to be significant factors in detecting suicidal crisis. Korean cultural maladaptation and academic maladaptation show specific relevance to suicidal crisis among international students studying in Korea. Although these factors are not directly related to suicide in general, we found that failure to adapt to culture and excessive stress in school can affect suicide crisis among international students.

This study’s limitations and suggestions for future studies are as follows. First, we focused on foreign students living in Korea and developed several additional questions specific to Chinese students, who account for the largest proportion of international students in Korea. Future efforts to derive additional questions reflecting the characteristics of other cultures should also be developed. Second, we used a self-report questionnaire, which risks social desirability bias and/or lack of introspection. Therefore, in future studies, it might be beneficial to develop a scale for significant others to rate the severity of suicide crisis. Third, about 65% of the study participants were found to have lived in Korea for less than 3 years, and close to 40% of them had a Korean level below intermediate. Because the period of residence in the international location and the level of the local language are variables closely related to acculturation, we look forward to expanding this research with respondents who have lived in Korea for a longer time and with a higher level of Korean. Fourth, with respect to data curation, we sought to obtain the necessary data from various sources, and performed data cleaning to ensure that the collected data was analyzable and reliable. For classification and organization, we divided the data into two categories: Chinese students and other international students. In future studies, as more data is accumulated, it may be possible to classify the data by each country’s international students. Additionally, if more data is gathered through study consortiums, we will undertake data integration to create a consistent dataset. Finally, we developed questions in Korean, English, and Chinese to facilitate participation by a wide range of nationalities. Nevertheless, there could have been students who could not comfortably use any of these three languages. Future research might consider conducting a survey in additional languages most familiar to the research participants.

## Data Availability

The raw data supporting the conclusions of this article will be made available by the authors, without undue reservation.

## References

[ref1] Baik J. S. (2011). Variables affecting college adaptation among international college students in Korea. J. Korean Home Manage. Assoc. 29, 119–131.

[ref2] Baik J. S. (2013). The influence of individuation of international college students in Korea on college adaptation and acculturative stress. Youth Facil Environ 11, 57–65.

[ref3] Baker R. W. Siryk B. (1989). Student adaptation to college questionnaire (SACQ). Los Angeles, CA: Western Psychological Services.

[ref4] Beck A. T. Kovacs M. Weissman A. (1979). Assessment of suicidal intention: the scale for suicide ideation. J. Consult. Clin. Psychol. 47, 343–352. doi: 10.1037/0022-006X.47.2.343 469082

[ref5] Buyadaa N. Yu K. L. (2021). The effect of acculturative stress on depression of Mongolians in Korea: focusing on moderating effect of social support. Korean Psychol J Culture Soc Issues 27, 35–49. doi: 10.20406/kjcs.2021.2.27.1.35

[ref6] Davidson C. L. Wingate L. R. Slish M. L. Rasmus K. A. (2010). The great black Hope: Hope and its relation to suicide risk among African Americans. Suicide Life-Threat 40, 170–180. doi: 10.1521/suli.2010.40.2.170, PMID: 20465352

[ref7] Ding X. Lee Y. W. Kim C. Y. (2021). A study on the recovery of sub-syndromatic depression in Chinese students. J Korea Multimedia Soc 24, 1425–1434. doi: 10.9717/kmms.2021.24.10.1425

[ref8] Gorsuch R. L. (1983). Factor analysis. 2nd Edn. Hillsdale, NJ: Lawrence Erlbaum.

[ref9] Han Y. K. (2009). Factors affecting school adjustment of international students in Korean universities: with a focus on Mongol students. Unpublished master’s thesis. Seoul: Soongsil University.

[ref10] Im S. B. Jung C. S. (2002). A comparison of stressors and coping behaviors of the high school students who have suicidal ideation vs. those who do not. J. Korean Acad. Nurs. 32, 254–264. doi: 10.4040/jkan.2002.32.2.254

[ref11] Jahng S. (2015). Best practices in exploratory factor analysis for the development of the Likert-type scale. Korean J. Clin. Psychol. 34, 1079–1100. doi: 10.15842/kjcp.2015.34.4.010

[ref12] Jeong H. S. (2012). Stress and counseling need of Chinese students in Korea. J Korean Data Anal Soc 14, 949–963.

[ref13] Jeong D. H. Shim E. J. (2022). Qualitative study on experiences of suicidal ideation and behavior among college students. Korean J Stress Res 30, 204–212. doi: 10.17547/kjsr.2022.30.4.204

[ref14] Jin H. Chi E. L. (2017). Development and validation of the dropout intention scale for Chinese students studying in Korean universities. J Edu Eval 30, 101–121.

[ref15] Jin L. Lim S. A. (2021). Structural relationship of Chinese student’s academic stress, loneliness, Korean language proficiency, self-efficacy and school dropout intention. J Learner-Centered Curri Ins 21, 705–717. doi: 10.22251/jlcci.2021.21.5.705

[ref16] Joeng J. R. Kim E. Y. Choi S. A. Lee Y. J. Kim J. K. (2015). The relation between stress of college life and suicidal ideation: mediating effects of perfectionistic concern over mistakes, social support, and depression. Korean J Couns Psychothera 27, 325–349.

[ref17] Joiner T. E. (2005). Why people die by suicide. Cambridge, MA: Harvard University Press.

[ref18] Kaiser H. F. (1974). An index of factorial simplicity. Psychometrika 39, 31–36. doi: 10.1007/BF02291575

[ref19] Kang H. C. (2013). A guide on the use of factor analysis in the assessment of construct validity. J. Korean Acad. Nurs. 43, 587–594. doi: 10.4040/jkan.2013.43.5.587 24351990

[ref20] Kang H. Y. Chang E. J. (2018). Relationship among failed belongingness, perceived burdensomeness, and suicidal ideation of psychiatric inpatients: mediation effect of depression. J Digital Converg 16, 461–469. doi: 10.14400/JDC.2018.16.2.461

[ref21] Keimyung University Student Counseling Center (2019). 2019 the need analysis report of international students. Daegu, Republic of Korea: Keimyung University.

[ref22] Kim S. Y. (2015). 85,000 international students… ‘emergency’ for psychological health university news network. Available at: http://news.unn.net/news/articleView.html?idxno=144261 (Accessed October 01, 2023).

[ref23] Kim J. Y. Chung Y. K. Lee J. S. (2009). The moderating effect of social supportive relationship in the contribution of adolescents’ experience of domestic child abuse to suicidal ideation. Korean J Soc Welfare Res 21, 119–144.

[ref24] Kim H. S. Kim B. S. (2008). Verification of the structural relationship model of suicidal ideation to its related variables. Korean J Couns Psychothera 20, 201–219.

[ref25] Kim K. S. Kim M. H. (2014). The influence of academic stress and acculturative stress of republic of Korean studying abroad on psychological adjustment. Korean J Culture Soc Issues 20, 67–88.

[ref26] Kim E. Y. Kim B. S. (2020). The mediating effects of hopelessness and the moderating effects of self-compassion and gender on the relationships between perceived burdensomeness, thwarted belongingness, and suicidal ideation in college students. Korean J Couns Psychothera 32, 287–313. doi: 10.23844/kjcp.2020.02.32.1.287

[ref27] Kim H. J. Sohn E. J. (2011). The effects of acculturative stress, ego-resilience and optimism on depression among Chinese students in Korea. Youth Facil Environ 9, 3–12.

[ref28] Kim Y. H. Yang M. S. Park H. R. (2015). A study on a relationship between university students life stress and suicidal ideation: mediating effect of mental health. J Digital Converg 13, 291–301. doi: 10.14400/JDC.2015.13.11.291

[ref29] Kwak Y. K. Lee B. J. Kim K. T. Koh M. K. Kim K. H. Kim G. C. (2021). The current status of international students and its policy implications in South Korea. Sejong, Republic of Korea: Korea Institute for Health and Social Affairs.

[ref30] Lee S. M. (1995). Factor analysis I. Seoul: Hakjisa.

[ref31] Lee S. J. (2008). A study on the hybridity of the reception of Korean popular culture: focusing on the Chinese students studying in the Busan region. Unpublished master’s thesis. Busan: National Korea Maritime & Ocean University.

[ref32] Lee Y. M. (2015). Experience of college students on suicide attempts. J. Korean Acad. Nurs. 45, 397–411. doi: 10.4040/jkan.2015.45.3.397 26159141

[ref33] Lee Y. M. Ha J. H. (2023). A qualitative research on the experiences of international student counselors who experienced suicide of a foreign student client: focusing on the university counseling centers. J Learner-Centered Curri Ins 23, 183–203. doi: 10.22251/jlcci.2023.23.11.183

[ref34] Lee Y. S. Kim E. H. (2015). Chinese international students’ types of acculturation, acculturative stress, and adjustment to college. Korean J School Psychol 12, 295–316. doi: 10.16983/kjsp.2015.12.3.295

[ref35] Lee H. W. Kwon M. J. Park C. M. (2023). Factors of foreign students’ health behavior and academic stress on depression. Health Welfare 25, 133–156. doi: 10.23948/kshw.2023.06.25.2.133

[ref36] Lee S. J. Lee J. E. (2021). A study on the mediating effect of autonomy, competence, relatedness in the relationship of acculturation stress to college adjustment among Chinese international students in Korea. J Learner-Centered Curri Ins 21, 485–502.

[ref37] Lee O. N. Park G. A. (2019). The moderating effect of bicultural competence on the relationship between acculturative stress and depression among international university students in Korea. Multicultural Edu Stud 12, 1–19. doi: 10.14328/MES.2019.3.31.1

[ref38] Lim S. R. Lee J. A. (2017). The effect of life stress, cultural adaptation stress and academic satisfaction in Chinese students studying in the Republic of Korea: target on beauty major college students. J Korea Acad-Indus Coop Soc 18, 189–199. doi: 10.5762/KAIS.2017.18.12.189

[ref39] Ministry of Education (2022). 2022 statistics of international students in universities and colleges in Korea. Sejong, Republic of Korea: Ministry of Education.

[ref40] Mun J. H. Yoon J. W. (2021). An analysis of the adaptation-related research trends of foreign students. J Humanities Soc Sci 12, 2931–2940. doi: 10.22143/HSS21.12.4.208

[ref41] Nguyen M.-H. Vuong Q.-H. Toan H. M. Le T.-T. (2021), Mindsponge mechanism. Available at: https://philpapers.org/rec/NGUMM (accessed July 29, 2023).

[ref42] O’Connor R. C. Nock M. K. (2014). The psychology of suicidal behavior. Lancet Psychiat 1, 73–85. doi: 10.1016/S2215-0366(14)70222-6 26360404

[ref43] Oh H. Y. Lee Y. H. (2018). Difficulties of international students recognized by professionals and psychological support plan. Korean Soc Wellness 13, 101–121. doi: 10.21097/ksw.2018.11.13.4.101

[ref44] Park C. M. (2022). The relationship between foreign student academic stress and depression tendency: the mediating effect of self-esteem. J Korean Soc Welfare Health Edu 23, 13–24. doi: 10.35133/kssche.20220531.02

[ref45] Park S. J. Kim J. N. (2017). The mediating effect of early maladaptive schemas in the relationship of interpersonal problems and suicidal ideation of university students. Korean J. Youth Stud. 24, 175–198. doi: 10.21509/KJYS.2017.03.24.3.175

[ref46] Park S. J. Kim J. N. (2018). The mediating effects of perceived burdensomeness and thwarted belongingness and the moderating effect of reasons for living on the relationship between depression and suicidal ideation in young adults. Korean J Couns Psychothera 30, 877–908. doi: 10.23844/kjcp.2018.08.30.3.877

[ref47] Park S. Y. Lee S. Y. (2021). The relationship among thwarted belongingness, meaning in life, and suicidal ideation of college students: the moderated mediating effect of extraversion. Korean J Applied Devel Psychol 10, 17–33. doi: 10.22839/adp.2021.10.3.17

[ref48] Pavlacic J. M. Weber M. C. Torres V. A. Ho L. Y. Buchanan E. M. Schulenberg S. E. (2023). Perceived meaning, pandemic self-efficacy, social support, and discrimination predict trajectories of peri-pandemic growth and distress for international students. Psychol Trauma 15, 469–473. doi: 10.1037/tra0001417, PMID: 36622727

[ref49] Poyrazli S. Kavanaugh P. R. Baker A. Al-Timimi N. (2004). Social support and demographic correlates of acculturative stress in international students. J. Coll. Couns. 7, 73–82. doi: 10.1002/j.2161-1882.2004.tb00261.x

[ref50] Radloff L. S. (1977). The CES-D scale: a self-report depression scale for research in the general population. Appl Psych Meas 1, 385–401. doi: 10.1177/014662167700100306

[ref51] Reynolds W. M. (1987). Suicidal ideation questionnaire (SIQ). Odessa, FL: Psychological Assessment Resources.

[ref52] Rosenberg M. (1965). Society and the adolescent self-image. Princeton, NJ: Princeton University Press.

[ref53] Sandhu D. S. Asrabadi B. R. (1994). Development of an acculturative stress scale for international students: preliminary findings. Psychol. Rep. 75, 435–448. doi: 10.2466/pr0.1994.75.1.435, PMID: 7809315

[ref54] Seong T. J. (2014). Educational evaluation. Seoul: Hakjisa.

[ref55] Shannon C. E. (1948). A mathematical theory of communication. Bell Sys Tech J 27, 379–423. doi: 10.1002/j.1538-7305.1948.tb01338.x

[ref56] Shin I. C. Han J. E. Park H. M. (2018). A study of the dropout rate of international students and its factors in South Korea. J Migration Soc 11, 105–133. doi: 10.14431/jms.2018.08.11.2.105

[ref57] Thomas M. Choi J. B. (2006). Acculturative stress and social support among Korean and Indian immigrant adolescents in the United States. J Sociol Soc Welfare 33, 123–145. doi: 10.15453/0191-5096.3164

[ref58] Van Orden K. A. Witte T. K. Cukrowicz K. C. Braithwaite S. R. Selby E. A. Joiner T. E. (2010). The interpersonal theory of suicide. Psychol. Rev. 117, 575–600. doi: 10.1037/a0018697, PMID: 20438238 PMC3130348

[ref59] Van Orden K. A. Witte T. K. Gordon K. H. Bender T. W. Joiner T. E. (2008). Suicidal desire and the capability for suicide: tests of the interpersonal-psychological theory of suicidal behavior among adults. J. Consult. Clin. Psychol. 76, 72–83. doi: 10.1037/0022-006x.76.1.72, PMID: 18229985

[ref60] Vuong Q. H. (2023). Mindsponge theory. Boston, MA: Sciendo.

[ref61] Wong Y. J. Koo K. Tran K. K. Chiu Y. C. Mok Y. (2011). Asian American college students’ suicide ideation: a mixed-methods study. J. Couns. Psychol. 58, 197–209. doi: 10.1037/a0023040, PMID: 21463030

[ref62] Xiao X. L. Ahn D. H. (2016). Influence of acculturative stress on psychological well-being and academic adjustment of Chinese international students in Korea. Korean Edu Inquiry 34, 133–155. doi: 10.22327/kei.2016.34.3.133

[ref63] Yoon M. A. Lee H. K. (2022). The moderated mediation effect of self-compassion and early maladaptive schemas between relationship of interpersonal problems and suicidal ideation. Korean J. Youth Stud. 29, 1–25. doi: 10.21509/KJYS.2022.08.29.8.1

[ref64] Yu J. X. Choo S. Y. Lim S. M. (2014). The relationships between acculturation, ethnic group and psychological well-being, depression of foreign students in Korea. Korean J Cult Soc Issues 20, 1–18.

[ref65] Zhou H. Kim S. Lee S. (2023). Effects of cultural adaptation stress on university life adaptation of foreign students in Korea: a modulating effect of self-efficacy. Culture Converg 45, 871–880. doi: 10.33645/cnc.2023.05.45.05.871

[ref66] Zimet G. D. Dahlem N. W. Zimet S. G. Farley G. K. (1988). The multidimensional scale of perceived social support. J. Pers. Assess. 52, 30–41. doi: 10.1207/s15327752jpa5201_2 2280326

